# Treatment Modifications After Drug Shortages Among Primary Care Physicians

**DOI:** 10.1001/jamanetworkopen.2025.52802

**Published:** 2026-01-07

**Authors:** Jennie B. Jarrett, Katlyn E. Dillane, Geoff Hollett

**Affiliations:** 1 American Medical Association, Chicago, Illinois

## Abstract

This cross-sectional study assesses the pervasiveness, changes to treatment and outcomes, and administrative burdens of drug shortages for primary care physicians and patients.

## Introduction

US drug shortages are prevalent due to supply chain disruptions, limited manufacturers, small profit margins, regulatory burdens, natural disasters, and demand surges.^1,2,3,4^ Research is limited on drug shortage, physician practice, and quality of care interactions. This study investigated the outcomes associated with drug shortages among primary care physicians (PCPs) and patients, including pervasiveness, treatment changes and outcomes, and administrative burdens.

## Methods

In this study, a cross-sectional, web-based survey was fielded from July to August 2024 to PCPs affected by drug shortages. The study was deemed exempt by the University of Illinois Chicago institutional review board because no identifying information from participants was obtained. Participants provided informed consent. Two-tailed *P* < .05 was considered statistically significant (SPSS Statistics, version 29, IBM Inc). Details on recruitment, survey development, and methods are in the eMethods in Supplement 1. Reporting follows the STROBE guideline.

## Results

Of 1281 people screened, 902 met inclusion criteria. Perceived prevalence of drug shortage in the past 6 months was 88% (795 of 902). Participants were from US family medicine practices (337 [42%]), outpatient-only practices (599 [75%]), urban settings (616 [78%]), and private practices (562 [71%]).

Outcomes associated with drug shortages were reported for 1 in 5 patients (mean [SD], 20% [17%]; median [IQR], 15% [10%-25%]) (Table 1). Drug categories with the highest rate of severe outcomes (or “impact” according to the survey question) were endocrinology (427 [54%]), stimulant (416 [52%]), infectious disease (210 [26%]), pulmonology (135 [17%]), and pain management (112 [14%]). Pediatricians perceived a larger impact associated with shortages of infectious disease (η^2^ = 0.12; *P* < .001), pulmonology (η^2^ = 0.04; *P* < .001), and stimulant (η^2^ = 0.14; *P* < .001) drugs.

**Table 1.** Drug Shortages Across Population, Frequency, Duration, and Outcomes

Variable	No. (%) of survey responses^a^ (N = 795)
Patient panel with drug shortage interaction, %	
Median (IQR)	15 (10-25)
Mean (SD)	20.2 (16.5)
Frequency of drug shortage interaction in practice	
Daily	240 (30)
Weekly	303 (38)
Monthly	135 (17)
Less often than monthly	117 (15)
Duration of drug shortage interaction, wk	
<1	31 (4)
1-4	279 (35)
5-12	241 (30)
>12	244 (31)
Patient outcomes and resultant action of drug shortages	
Discontinued medication	786 (98)
Minor adverse event	456 (57)
Disease progression	393 (49)
Patient visited another country for medication or used compounding pharmacy	393 (49)
Patient substituted a different supplement	276 (35)
Patient consulted a nonphysician practitioner	218 (27)
Major adverse event	103 (13)
Patient death	19 (2)
Other	48 (6)

^a^ Unless otherwise indicated.

Drug shortages were associated with changes in the quality of care for 691 PCPs (87%). In practice modification, altering the drug of choice was more common (732 [92%]) than postponing prescribing (502 [63%]). Pediatricians were less likely to postpone prescribing compared with other groups (pediatrics mean [SD]: 2.47 [0.86] vs family medicine: 2.21 [0.79] vs internal medicine: 2.19 [0.83]; analysis of variance *P* < .001 across groups).

Drug shortage management considerations were drug efficacy (709 [89%]), adverse effects (646 [81%]), out-of-pocket price (617 [78%]), prior authorization (582 [73%]), and administration route (459 [58%]). Additionally, 349 participants (44%) reported concerns that there may be no substitution available, 241 (30%) described requiring a combination of substitute medications, and 85 (11%) considered dispensing errors from changing treatment.

Physicians most often were notified about drug shortages from patients or community pharmacists (Table 2). Administrative burdens included prior authorization (73 [9%] always, 593 [75%] sometimes, 109 [14%] rarely, and 20 [3%] never), overtime (90 [11%] always, 384 [48%] sometimes, 233 [29%] rarely, and 88 [11%] never), frustration (461 [58%] always, 283 [35%] sometimes, 48 [6%] rarely, and 4 [1%] never), and burnout (148 [19%] significantly, 305 [38%] moderately, 300 [38%] slightly, and 42 [5%] never). To cover increased workload, respondents required a median (IQR) of 0.5 (0.25-1.0) full-time equivalents of new staff time.

**Table 2.** Administrative Responses to Drug Shortages

Physician notification mechanism for a drug shortage^a^	No. (%) of survey responses	
	When a drug shortage occurs	When a drug shortage is resolved
Community pharmacist	554 (70)	453 (57)
Patient communication	580 (73)	482 (61)
Health system administration notification	163 (21)	136 (17)
Professional or organizational society	161 (20)	93 (12)
Online resource	150 (19)	112 (14)
Government resources (FDA)	109 (14)	83 (10)
EHR	112 (14)	77 (10)
Clinical pharmacist in practice	193 (24)	173 (22)
Other physicians in practice	298 (37)	247 (31)
Other health care staff in practice	171 (22)	144 (18)
Other	12 (2)	28 (4)

Abbreviations: EHR, electronic health record; FDA, US Food and Drug Administration.

^a^ Health system physicians vs private practice were more likely to hear from an administrator when a drug shortage occurred (45 of 150 [30%] vs 118 of 645 [18%], *P* = .001) or resolved (34 of 150 [23%] vs 102 of 645 [16%], *P* = .04) and more likely to hear from clinical pharmacists in their practice about occurrence (53 of 150 [35%] vs 140 of 645 [22%], *P* < .001) or resolution (44 of 150 [29%] vs 129 of 645 [20%], *P* = .01). Of note, the n for private practice here is from the screening Yes/No question for inclusion (n = 645) versus the demographic n for private practice (n = 562) was determined from the demographic Select All That Apply question.

## Discussion

This study highlights the high perceived prevalence of US drug shortages and negative outcomes related to patient care, primary care practice, and physician well-being. Drug shortage communication channels are lacking. Evidence-based resources or interventions, such as electronic health record alerts, were substituted for direct patient information, presumably when patients did not fill their prescriptions. This limits the ability to consider alternative treatments, may undermine a patient’s trust in their physician, and may contribute to patients discontinuing care or seeking riskier alternatives.

New care plan formulations caused by drug shortages are challenging for physicians, with multiple factors considered to ensure appropriate care alternatives and an increased staff workload with limited reimbursement. Drug shortages result in more prior authorization requests because alternate medications may differ from the standard of care or may not have included a patient’s formulary.^5,6^ This workplace stress caused most participants to feel frustrated by drug shortages. Study limitations include overestimation of the overall perceived prevalence due to a potential for oversampling of physicians experiencing and impacted by drug shortages, residual confounding, potential lack of disclosure for patients and physicians, and the challenge to prove the response was due to a true drug shortage.

## Notes

Supplement 1. **eMethods.** Survey Methodology

Supplement 2.
Data Sharing Statement
