# Supplementary material for: Treatment Modifications After Drug Shortages Among Primary Care Physicians
Source: JAMA Netw Open. 2026 Jan 7;9(1):e2552802. doi: 10.1001/jamanetworkopen.2025.52802 (PMC12780924; doi:10.1001/jamanetworkopen.2025.52802)
Supplement: Supplement 1. — eMethods. Survey Methodology [file jamanetwopen-e2552802-s001.pdf]

Supplemental Online Content

Jarrett JB, Dillane K, Hollett G. Treatment modifications after drug shortages among primary care physicians. *JAMA Netw Open*. 2026;9(1):e2552802. doi:10.1001/jamanetworkopen.2025.52802

eMethods. Survey Methodology

This supplemental material has been provided by the authors to give readers additional information about their work.

© 2026 Jarrett JB et al. *JAMA Network Open.*

eMethods. Survey Methodology

A cross-sectional survey was developed and conducted to study the impact of drug shortages on primary care physicians and their practice in the United States. This survey study was granted exemption by the University of Illinois Chicago Institutional Review Board. Participants were electronically recruited from a panel of physicians maintained by Dynata® (2024) between July and August 2024.

Sample

The analytic sample included physicians board-certified in internal medicine, family medicine, pediatrics, or internal medicine-pediatrics medical specialties whoprovide outpatient primary care for the majority of their practice. Respondents from outside the United States were excluded. We recruited respondents from private and health system practices, with health system practice capped at 50% of the total sample to ensure adequate private practice sampling. Respondents were from both rural and urban geographic areas, with quotas in place to ensure at least 20% of respondents came from rural areas to match United States Census data.[15] Finally, to be eligible to complete the full survey, participants needed to endorse that any drug shortage has impacted their practice in the last six months. Respondents were provided the Food and Drug Administration definition of a drug shortage: "A period of time when the demand or projected demand for the drug within the United States exceeds the supply of the drug" for reference.[16] Dynata® (2024) compensated online respondents at standard rates.

© 2026 Jarrett JB et al. *JAMA Network Open.*

Questionnaire

Survey questions were developed based on an extensive literature review of the current drug policy landscape and pilot-tested by a working group of physician stakeholders. Primary outcomes were the perceived prevalence of drug shortages, impact of drug shortages on primary care patients, the frequency of impact, the specific drug categories impacted by shortages (informed by the FDA Drug Shortages list in April 2024), and impact on physician practice administration and wellbeing. Survey questions included measures of the percentage of respondents' patients impacted by a drug shortage, the frequency of which shortages impacted physicians' practice, and the severity of impact across 13 drug categories based on the American Society of Health System Pharmacists Drug Shortage Bulletin.[17] We included questions to assess administrative burden and practice actions to mitigate the impact of drug shortages. To determine the impact of drug shortages on patient care, we developed 4-point Likert scale questions assessing how often physicians felt the quality of the care was impacted, how often patients discontinue or improperly took their medications due to a shortage, and how often physicians were forced to postpone prescribing or altering their treatment plans due to a shortage. Physicians were also asked to select which patient outcomes they observed due to a shortage and to select what factors they consider in their medical decision-making when substituting a medication due to a drug shortage.

The study followed the Strengthening the Reporting of Observational Studies in Epidemiology (STROBE) reporting guidelines.[18] The finalized survey with all questions is included as a supplementary document to this submission.

© 2026 Jarrett JB et al. *JAMA Network Open.*

Analysis

Data analysis was performed using IBM SPSS Statistics (Version 29). Two-tailed $p<0.05$ was considered statistically significant for all tests. We conducted descriptive statistics for study variables and physician characteristics. Independent samples two-tailed T-tests were used to analyze the differences between two groups, and 1-way ANOVA tests were used for variables with three or more groups with post-hoc Bonferroni corrections. Cohen's d and eta-squared ($\eta 2$) calculations were used to demonstrate effect size.

© 2026 Jarrett JB et al. *JAMA Network Open.*

Appendix 1. Survey

Screening Questions for Participant Inclusion (identifying primary care physicians across urban and rural areas, as well as private practice and health-system practices).

	Question	Response Options
Screener 1	What is your board-certified medical specialty?	Acceptable answers:<br>• Internal medicine<br>• Family medicine<br>• Pediatrics<br>• Internal medicine/pediatrics<br>• Preventative medicine<br>• I'm not board certified - terminate<br>Terminate all other specialties
Screener 2	Do you provide primary care for the majority of your practice?	• Yes<br>• No - Terminate
Screener 3	What state do you practice in?	[Drop down of all US states and territories + "I don't live in the United States" – terminate if outside US]
Screener 4	What is your primary practice setting?	• Inpatient - terminate<br>• Outpatient<br>• Both, majority inpatient – terminate<br>• Both, majority outpatient
Screener 5	Do you work in a private practice?	• Yes<br>• No – Terminate once 50% threshold reached
Screener 6	What kind of geographic area do you practice in?	• Urban (An area of >50,000 people) – Terminate once 75% threshold reached to ensure 25% rural responses<br>• Rural (all other areas)
Screener 7	In the last 6 months, has any drug shortage impacted your practice?<br>*FDA Definition of a drug shortage: "A period of time when the demand or projected demand for the drug within the United States exceeds the supply of the drug/"*	• Yes<br>• No - Terminate

© 2026 Jarrett JB et al. *JAMA Network Open.*

START OF THE SURVEY:

Prevalence Questions

Instructions: Consider over the last 6 months:

1	Question	Response Type
1.1	What percentage of your patients were impacted by a drug shortage, on a scale of 0 (none) to 100 (all patients)?	0-100 slider
1.2	How often do drug shortages impact your practice as a physician?	• Daily<br>• Weekly<br>• Monthly<br>• Greater than monthly

Question 1.3

For each of the following drug classes, please rate the severity of the impact on your practice. Please select one option.

Drug Classes	Major	Minor	No impact
Antidepressants/Anxiolytic (Examples: Duloxetine, Lorazepam)	☐	☐	☐
Cardiology (Examples: Quinapril, Losartan)	☐	☐	☐
Dermatology (Examples: Fluocinolone, Tretinoin)	☐	☐	☐
Endocrinology (Examples: Semaglutide, Insulin Aspart)	☐	☐	☐
Gastroenterology (Examples: Dicyclomine, Magnesium Citrate)	☐	☐	☐
Hormonal Agents (Examples: Testosterone, Estradiol)	☐	☐	☐
Infectious Disease (Examples: Liquid Amoxicillin, Penicillin G Benzathine)	☐	☐	☐
Oncology (Examples: Mycophenolate, Mercaptopurine)	☐	☐	☐
Ophthalmology (Examples: Ofloxacin, Timolol)	☐	☐	☐
Pain management (Examples: Hydrocodone, Oxycodone)	☐	☐	☐
Pulmonology (Examples: Budesonide, Mometasone)	☐	☐	☐
Rheumatology/Immunology (Examples: Sulfasalazine, Belimumab)	☐	☐	☐
Stimulants/Attention Deficit Hyperactivity Disorder drugs (Examples) Methylphenidate, Amphetamine Salts)	☐	☐	☐
Other (Write in: _________)	☐	☐	☐

© 2026 Jarrett JB et al. *JAMA Network Open.*

1.4	In the last 6 months, how long did a drug shortage typically impact your practice?	• Less than 1 week<br>• Between 1 and 4 weeks<br>• Between 5 and 12 weeks<br>• Greater than 12 weeks
1.5	Think about the longest drug shortage you've experienced in the last 5 years. About how many months did it last?	[Drop down numerical responses: 1 month, 2 months, etc., cut-off >24 months]

Patient Care Questions

Instructions: Consider the last 6 months and select one response.

2	**Question**	**Response Type**			
		Often	Sometimes	Rarely	Never
2.1	In your opinion, do drug shortages impact the **quality** of patient care you deliver?	☐	☐	☐	☐
2.2	How often do patients **not take or discontinue** medications due to a drug shortage?	☐	☐	☐	☐
2.3	How often do you **postpone prescribing medication** due to a drug shortage?	☐	☐	☐	☐
2.4	How often do you **alter your treatment medication of choice** due to a drug shortage?	☐	☐	☐	☐

	**Question**	**Response Options**
2.5	What patient outcomes and/or actions have you seen due to a drug shortage? Please select all that apply.	☐ Major adverse event requiring hospitalization<br>☐ Minor adverse event or side effect<br>☐ Progression of disease<br>☐ Death<br>☐ Patient initiated going to an alternative, non-physician provider (ex. Chiropractor, naturopath)<br>☐ Patient self-initiated taking supplement, herbal product, or compounded agent<br>☐ Obtaining medications from another country<br>☐ None<br>☐ Other (Write in: ____________)

© 2026 Jarrett JB et al. *JAMA Network Open.*

2.6	When substituting a medication due to a drug shortage, what are considerations you make of the alternative medication? Please select all that apply.	☐ Efficacy<br>☐ Side effects<br>☐ Administration routes/frequency<br>☐ Prior authorization requirement or lack of coverage<br>☐ Out of pocket price<br>☐ No appropriate substitution available<br>☐ Combination of medications needed for substitution<br>☐ Dispensing errors due to shortages<br>☐ Other (Write in: _________)

Administrative Questions

3	**Question**	**Response Options**
3.1	How do you **find out that a drug is in shortage**? Please select all that apply.	☐ Community pharmacist<br>☐ Patient<br>☐ Health system administrator<br>☐ Professional or organizational society<br>☐ Online Resource<br>☐ Government Resource (FDA)<br>☐ Electronic Health Record<br>☐ Clinical pharmacist in your practice<br>☐ Other physicians<br>☐ Other health care staff in your practice<br>☐ Other (Please specify: ___________)
3.2	How do you **find out when a drug shortage is resolved?** Please select all that apply.	☐ Community pharmacist<br>☐ Patient<br>☐ Health system administrator<br>☐ Professional or organizational society<br>☐ Online Resource<br>☐ Government Resource (FDA)<br>☐ Electronic Health Record<br>☐ Clinical pharmacist in your practice<br>☐ Other physicians<br>☐ Other health care staff in your practice<br>☐ Other (Please specify: ___________)

© 2026 Jarrett JB et al. *JAMA Network Open.*

3.3	What practice actions occur during a drug shortage? Please select all that apply.	☐ Automatic drug substitutions<br>☐ Modifying prescribing patterns<br>☐ Limiting prescriptions to certain indications<br>☐ Other (Write in: _________)

Please select one of the following responses:

	Question	Response Type			
		Always	Sometimes	Rarely	Never
3.4	When substituting to another drug or medication, how often have you had to fill out prior authorization forms?	☐	☐	☐	☐
3.5	How often did you or your clinical care team stay late or work overtime due to a drug shortage?	☐	☐	☐	☐
3.6	How often are you frustrated by drug shortages?	☐	☐	☐	☐

3.7	Which of your staff work on mitigating drug shortages? Please select all that apply.	☐ You<br>☐ Nursing Staff<br>☐ Clinical pharmacists in your practice<br>☐ Behavioral health or social work<br>☐ Office staff<br>☐ Other MDs in your practice<br>☐ Other (Please specify: ___________)
3.8	How much staff FTE (full-time equivalent) did you need to increase to adequately respond to drug shortages? Please answer to the closest tenth (ex. 4.2 FTE)<br><br>1 FTE = 40 hours per week	[Fill in blank]
3.9	How do drug shortages contribute to your workplace stress/burnout?	☐ Significantly<br>☐ Moderately<br>☐ Slightly<br>☐ Never

© 2026 Jarrett JB et al. *JAMA Network Open.*

Wrap up

4.1	How can the American Medical Association support you regarding drug shortages?	[Qualitative text box free response]
4.2	Is there anything else you'd like to add regarding drug shortages?	[Qualitative text box free response]

Demographics

Demo 1	What is your practice type? Please select all that apply.	☐ Private practice<br>☐ Health system practice<br>☐ Federally qualified health center<br>☐ Indian Health Service<br>☐ Community-based clinic<br>☐ Veteran's health administration<br>☐ Other (Specify: ___________)

© 2026 Jarrett JB et al. *JAMA Network Open.*
