# Supplementary material for: Treatment Modifications After Drug Shortages Among Primary Care Physicians
Source: JAMA Netw Open. 2026 Jan 7;9(1):e2552802. doi: 10.1001/jamanetworkopen.2025.52802 (PMC12780924; doi:10.1001/jamanetworkopen.2025.52802)
Supplement: Supplement 2. — Data Sharing Statement [file jamanetwopen-e2552802-s002.pdf]

Data Sharing Statement

Jarrett. Treatment Modifications After Drug Shortages Among Primary Care Physicians. *JAMA Netw Open*. Published January 07, 2026. doi:10.1001/jamanetworkopen.2025.52802

Data
Data available: No
